# Enhancing Localization Accuracy and Reducing Processing Time in Indoor Positioning Systems: A Comparative Analysis of AI Models

**DOI:** 10.3390/s25020475

**Published:** 2025-01-15

**Authors:** Salwa Sahnoun, Rihab Souissi, Sirine Chiboub, Aziza Chabchoub, Mohamed Khalil Baazaoui, Ahmed Fakhfakh, Faouzi Derbel

**Affiliations:** 1 Laboratory of Signals, Systems, Artificial Intelligence and Networks (SM@RTS), Digital Research Center of Sfax (CRNS), Sfax University, Sfax 3021, Tunisiarihab.souissi@stud.htwk-leipzig.de (R.S.); sirin.chiboub@ieee.org (S.C.); chabchoubaziza1@gmail.com (A.C.); mohamed_khalil.baazaoui@stud.htwk-leipzig.de (M.K.B.);; 2 National School of Electronics and Telecommunications of Sfax, Sfax 3018, Tunisia; 3 Smart Diagnostic and Online Monitoring, Leipzig University of Applied Sciences, Wächterstraße 13, 04107 Leipzig, Germany

**Keywords:** indoor positioning systems, AI, neural networks, ANN, RNN, LSTM, Kalman filter, RSSI measurements, ICM-20948 sensor

## Abstract

This paper presents a comparative study of different AI models for indoor positioning systems, emphasizing improvements in localization accuracy and processing time. This study examines Artificial Neural Networks (ANNs), Long Short-Term Memory (LSTM), Recurrent Neural Networks (RNNs), and the Kalman filter using a real Received Signal Strength Indicator (RSSI) and 9-axis ICM-20948 sensor. An in-depth analysis is provided in this paper for data cleaning and feature selection to reduce errors for all the models. We evaluate these models in terms of localization accuracy and prediction time. The RNN model shows the best performance, achieving a localization error of 0.247 m with a delay of 0.077 s per position location for an area of 12 m × 9.5 m using four anchors. This research highlights the importance of selecting AI models for effective mobile tracking according to test and validation data.

## 1. Introduction

Wireless sensor networks (WSNs) have been effective in monitoring, tracking, and controlling space, especially in environments where wiring is impractical or expensive [1]. Indoor positioning systems (IPSs) are essential for tracking individuals, objects, or devices within enclosed spaces. These systems are vital in various sectors, including industrial operations, healthcare, and navigation services, due to their ability to provide accurate and reliable location information [2]. IPS technology enhances operational efficiency and productivity in dynamic environments like factories and warehouses. It offers significant benefits such as improved logistics, faster decision-making, and effective inventory management. For instance, real-time knowledge of employees and asset locations can streamline production processes, reduce search time, and minimize manual checks [3].

Despite advancements, indoor positioning faces challenges like signal interference and the dynamic nature of indoor environments [4]. Traditional technologies like the Global Positioning System (GPS) are less effective indoors due to obstacles that obstruct line-of-sight transmission [5]. To address these issues, alternative techniques such as Received Signal Strength Indicator (RSSI) [6], Angle of Arrival (AOA) [7], Time of Arrival (TOA) [8], and Time Difference of Arrival (TDOA) have been developed to enhance accuracy and precision within indoor spaces [9].

The RSSI technique has several advantages. It is relatively cost-effective to implement as it relies primarily on existing hardware such as Wi-Fi, Bluetooth, or other wireless communication systems that already have signal strength information available. This eliminates the need for specialized sensors or expensive infrastructure. RSSI can be used for real-time tracking of objects or people within the convergence area, which is beneficial for applications such as asset tracking, personnel monitoring, or smart building systems [4].

Recent research has focused on integrating machine learning algorithms into IPSs to achieve higher accuracy. Algorithms like Artificial Neural Networks (ANNs) and Long Short-Term Memory (LSTM) [10] have shown promise in optimizing indoor positioning accuracy. However, the Kalman filter combines noisy sensor measurements with dynamic model predictions to provide refined position estimates over time [11]. The advancement of machine learning and deep learning technologies in indoor localization systems has made significant progress, but real-time evaluation remains a significant challenge.

Simulations or offline testing could be an effective solution before real implementation to minimize complexity and improve applications [12]. The offline phase of system evaluation is critical to ensure effective real-time implementation, especially for dynamic mobility tasks. Latency is a critical factor affecting system performance in various tasks. Traditional RSSI-based localization algorithms often fail in changing and noisy environments [13]. In contrast, integrating IMU sensors provides an essential solution for real-time tracking accuracy. This paper proposes a novel approach that combines RSSI with a 9-axis ICM-20948 sensor to improve mobile target tracking. To optimize the tracking performance, we conducted a comprehensive comparative study of various algorithms, including Artificial Neural Networks (ANNs), Recurrent Neural Networks (RNNs), Long Short-Term Memory (LSTM) networks, and the Kalman filter. Our purpose is to minimize computational time while maximizing accuracy, thereby improving the accuracy of real-time localization.

This paper is organized as follows: Section 2 reviews related works on indoor positioning systems and AI models. Section 3 explains the use of neural networks, including model structures for localization. Section 4 describes the Kalman filter and its role in enhancing positioning accuracy. Section 5 details the system setup and the experimental environment, including cross-validation and data processing methods. Section 6 outlines the proposed system, integrating AI models and filters. Section 7 presents the results, followed by a discussion of the system’s performance.

## 2. Related Works

Indoor positioning is widely used in many fields, such as healthcare [14], IoT systems [15], and industry [16] to track objects and monitor spaces, providing context-aware services that enhance efficiency and user experience. The field of indoor localization has evolved in recent years with the introduction of various technologies. Despite the growing interest, numerous challenges and drawbacks need to be addressed to develop more accurate and sustainable systems. The following Table 1 provides a summary of techniques used for indoor positioning, detailing the technologies employed and their performance characteristics.

In [17], the authors explore methods to enhance indoor localization accuracy using Bluetooth Low Energy (BLE) technology. The study evaluates several approaches, including multichannel transmission, RSSI signal aggregation, and the use of Feed-Forward Neural Networks (FNNs) for positioning. Experiments conducted in complex indoor environments revealed that combining multichannel transmission with RSSI aggregation significantly improved accuracy, reducing positioning errors by about 35% (from 1.5 m to 1 m). The use of FNNs, particularly in machine learning-assisted approaches, further refined the estimation of tag positions, although the performance varied depending on the specific combination of techniques used. This research offers valuable guidelines for the optimization of BLE-based localization systems tailored to specific accuracy requirements.

The paper [18] compares the effectiveness of K-nearest neighbor (KNN) and trilateration methods for indoor localization using RSSI data for three wireless technologies: ZigBee, Bluetooth Low Energy (BLE), and Wi-Fi. The study concludes that KNN outperforms trilateration, particularly in terms of accuracy and stability, with the optimal K-value determined to be 3 to avoid overfitting or underfitting. Wi-Fi emerges as the most reliable technology among the three, providing the best performance in indoor localization. The research highlights the limitations of trilateration in complex indoor environments and emphasizes the advantages of using KNN for more accurate and stable localization results. In [19], the authors proposed a method for proximity estimation using BLE RSSI and UWB range data, using a machine learning (ML) algorithm to improve indoor positioning accuracy. The study trained a Random Forest model with UWB data to predict distances based on BLE RSSI measurements. The model was evaluated in different indoor environments, and demonstrated its effectiveness in estimating proximity with a mean error of approximately 1 m for distances up to 5 m. The research highlighted the limitations of BLE-only systems and the potential of integrating UWB data to enhance positioning accuracy. The authors in [20] compare RSSI-based indoor localization methods using ZigBee networks. The study evaluates real and synthetic fingerprint datasets with algorithms such as K-nearest neighbor (KNN), multilayer perceptron (MLP), and Long Short-Term Memory (LSTM) networks in a controlled lab setting. The results show that real fingerprinting data, particularly with the MLP algorithm, provides the highest accuracy. The study also introduces the Error-to-Active Area (E/A) ratio for better accuracy assessment, highlighting the trade-offs between installation complexity and localization accuracy.

In [21], the authors investigate a Pedestrian Dead Reckoning (PDR) algorithm using an inertial measurement unit (IMU) for robust pedestrian tracking, even during trajectory changes. To enhance tracking accuracy, the study integrates four Ultra-Wideband (UWB) base stations with handheld UWB mobile tags equipped with ICM-20948 IMU sensors. Experiments were conducted in two distinct environments, including an office space measuring 15 m × 6 m. The UWB system demonstrated an error range of 0.1 to 0.5 m. Despite offering improved accuracy, the initial deployment of UWB technology incurs high costs. This study focuses exclusively on evaluating positional error, excluding considerations of delay. In [22], the authors introduce a novel indoor positioning method leveraging machine learning (ML) by fusing motion and ambient sensor data without relying on external infrastructure. The research presents the Motion-Ambient dataset, containing multivariate time series data, and models the problem as a multivariate time series classification (MTSC) task. ML architectures including DT, RF, LSTM, BiLSTM, CNN-1D, CNN-2D, MLP, and FCN are formulated, trained, and evaluated based on accuracy, loc-score, memory footprint, inference latency, and throughput. The models achieve accuracies exceeding 80%, meeting latency requirements for practical applications. Among them, CNN-1D offers the most balanced performance, achieving a latency of 0.05 ms and an accuracy of 0.93.

These works collectively highlight the advances in indoor positioning technologies, showcasing diverse applications and innovative approaches to enhancing localization accuracy and utility. Each study contributes to the broader understanding and implementation of effective indoor positioning systems across various use cases. In [4], we presented an algorithm for localizing mobile targets in an indoor wireless sensor network using the Wake-up Media Access Control protocol. We demonstrated that the collected RSSI values are significantly affected by various environmental factors such as obstacles, temperature, and humidity. To address this, we proposed the use of a Cauchy distribution to fit the simulated RSSI values to the measured ones. Despite this, the error in the estimated location of the mobile target remains high (over 2 m). For this reason, we have turned to AI models to improve the accuracy of target position estimation. In this paper, we provide a comparative analysis of AI models to develop a system that provides both high accuracy and reduced processing time. We focus on minimizing the error and processing time of the offline phase to ensure a faster localization method in real-time implementation. A combination of RSSI and 9-axis sensor is combined to maximize the accuracy of tracking a mobile target. A deep analysis of data cleaning, feature selection, and hyperparameter tuning of each model is treated to minimize the error and the time processing. The entirety of this study is based on real data measurements.

## 3. Neural Network

Deep learning is increasingly being used to process time series data due to its ability to handle complex temporal dependencies and improve prediction accuracy. Traditional methods often struggle with the volatile nature of the data, but deep learning techniques excel at automatically learning complex mappings between input and output sequences. This approach is useful in applications as diverse as image processing, speech recognition, and indoor positioning. Deep learning models can automatically extract important features and improve system efficiency. However, they require extensive training time, especially for large datasets [23]. The models begin with the input layer, which feeds data into multiple hidden layers, which are fully connected to the preceding layer. The hidden layers process the data, which are then passed to the output layer. The connections are depicted with arrows in the figure below, demonstrating how input nodes are connected to hidden layers, and how hidden layers feed into the output layer to produce the final results. This schematic highlights the sequential data flow and the overall structure of the network.

### 3.1. Artificial Neural Networks: ANNs

**Figure 1:**
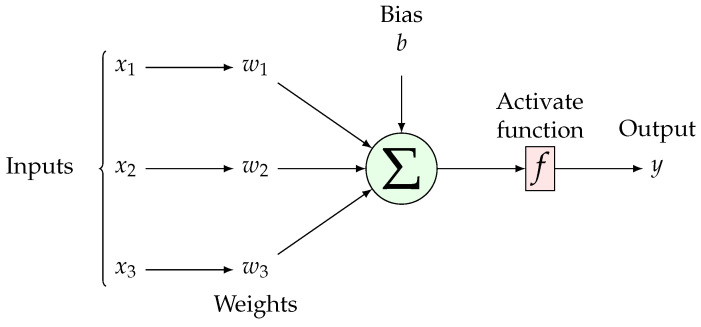
Schematic of a neural network unit showing inputs, weights, activation function, and output.

An ANN is used in a number of classification and prediction scenarios. Figure 1 depicts the system’s structure and functionality For localization, the ANN must be trained using the feature values and corresponding coordinates obtained during the offline phase. Once trained, the ANN can be used to determine the user’s location based on the online RSSI measurements. The neurons are modeled as weights where the strength of the connection is represented by the value of the weight. The activity of the neuron is divided into two components: an adder that sums all weighted inputs and an activation function that controls neuron output amplitude [24].

### 3.2. Recurrent Neural Networks (RNNs)

**Figure 2:**
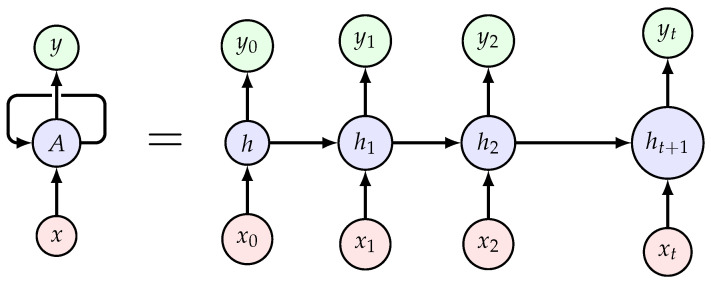
Recurrent Neural Network (RNN) scheme.

A Recurrent Neural Network (RNN) is a type of Artificial Neural Network in which the output is influenced not only by the current input, but also by historical data. RNNs are particularly effective for data with sequential dependencies, such as time series or spatial trajectories. For example, in indoor localization, a user’s current position is related to their previous positions due to the continuous nature of their movement. RNNs exploit this sequential relationship by using past measurements and trajectory information to improve localization accuracy. Figure 2 presents the scheme of an RNN. Essentially, an RNN works by processing sequences of data, allowing it to account for temporal dependencies in its predictions. An RNN is a function $f_{\theta }$ defined by (1), and the output is presented by Equation (2) [25]:

(1) $$h_{t}=\sigma_{h}\left(W_{xh}h_{t}+W_{hh}h_{t-1}+b_{h}\right)$$

(2) $$y_{t}=\sigma_{y}\left(W_{hy}h_{t}+b_{y}\right)$$

where $h_{t}$ represents the output at timestep *t*. $\sigma_{h}$ denotes the activation function (e.g., sigmoid or tanh). $W_{xh}$ is the weight matrix connecting input features to the hidden state. $W_{hh}$ is the weight matrix connecting the previous hidden state to the current hidden state. $h_{t-1}$ is the hidden state from the previous timestep. $b_{h}$ is the bias term.

### 3.3. Long Short-Term Memory (LSTM)

**Figure 3:**
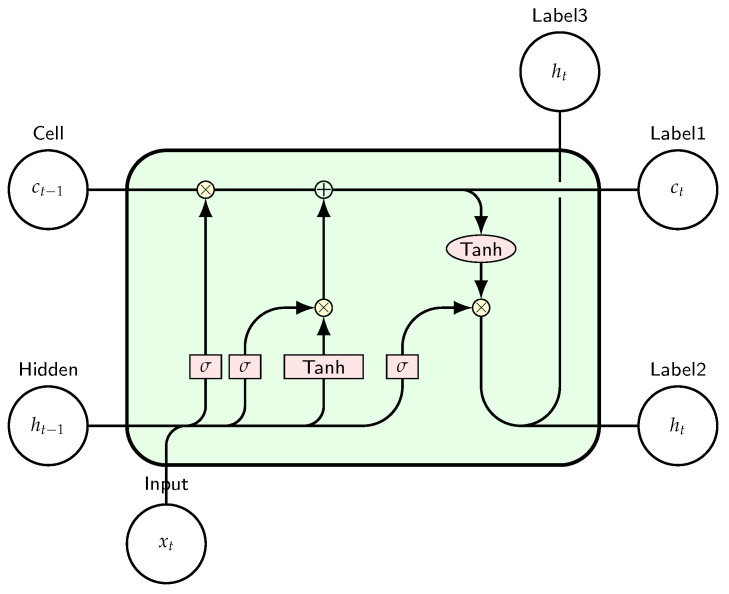
Long Short-Term Memory (LSTM) cell.

The Long Short-Term Memory (LSTM) network is a specialized type of Recurrent Neural Network (RNN) designed to address the problem of disappearing gradients. While a traditional RNN processes sequential data through its network-based memory and feedback loops, it often struggles to retain information over long sequences. LSTM networks enhance this capability by incorporating mechanisms that allow them to maintain gradients over a long period of time, thereby improving their ability to capture long-term dependencies in sequential data [26]. The LSTM cell diagram in Figure 3 illustrates the complex interactions between the different gates (forget, input, and output) and functions (sigmoid and tanh) used to manage the flow of information and maintain long-term dependencies. This structure helps to overcome the vanishing gradient problem common in standard RNNs, allowing the network to learn long-term relationships in sequences.

## 4. Kalman Filter

The Kalman filter (KF) is a recursive optimal estimator used to analyze time series data, originally based on linear transformations but extendable to nonlinear systems. The KF operates in two main steps: prediction and updating.

In the prediction step, the filter estimates the current state based on previous state estimates. This step generates a prediction, known as the prior estimate, because it relies on previous estimates without incorporating current observations.

In the update step, the prior estimate is adjusted by incorporating the current observations to refine the estimates of the current and next states. These two steps, prediction followed by updating, alternate continuously: prediction is performed until the next observation is available, followed by updating with the current observations [27]. The accelerometer measures linear acceleration along the x and y axes to detect motion, while the gyroscope measures angular velocity around these axes to track rotation and estimate orientation changes. By integrating accelerometer and gyroscope values into these matrices, the Kalman filter balances predicted states with noisy measurements to refine position, velocity, and orientation estimates [28]. Equations (3)–(6) describe the basic Kalman filter, assuming linear models for state transitions and observations:

(3) $$x_{k}=F_{k-1}x_{k-1}+W_{k-1}$$

(4) $$z_{k}=H_{k}x_{k}+V_{k}$$

where the following are denoted:

- $F_{k-1}$ is the state transition matrix;
- $H_{k}$ is the observation model matrix;
- $W_{k-1}$ represents process noise with covariance $Q_{k}$;
- $V_{k}$ represents measurement noise with covariance $R_{k}$.

The prediction step of the Kalman filter provides estimates of the next state and the associated error covariance:

(5) $${\hat{x}}_{k}^{-}=F_{k-1}{\hat{x}}_{k}$$

(6) $$P_{k}^{-}=F_{k-1}P_{k-1}^{+}F_{k-1}^{T}+Q_{k}$$

where the following are denoted:

- ${\hat{x}}_{k}^{-}$ is the predicted state estimate;
- $P_{k}^{-}$ is the predicted error covariance;
- ${\hat{x}}_{k}$ is the state estimate at time *k*;
- $P_{k-1}^{+}$ is the error covariance at time k−1;
- $Q_{k}$ is the process noise covariance matrix.

## 5. System Setup

The prototype setup for collecting real data consists of four anchors equipped with Spirit1 transceivers and one target equipped with both a Spirit1 transceiver and an ICM-20948 sensor. The hardware specifications are presented in the following subsections.

### 5.1. ICM 20948

The ICM-20948 is a 9-axis inertial measurement unit (IMU) sensor that integrates accelerometers, gyroscopes, and magnetometers to measure motion and orientation. For indoor positioning, the accelerometer tracks changes in velocity along three axes to determine motion and trajectory, while the gyroscope measures angular velocity about these axes to provide insight into the orientation and rotational motion of the device, which helps to estimate heading or direction. The magnetometer measures the strength and direction of the magnetic field, providing orientation relative to the Earth’s magnetic field. This is critical for improving positioning accuracy indoors where GPS signals are limited. Table 2 details the specifications of the IMU components. For the accelerometers, the full-scale range is ±2, 4, 8, and 16 g with resolutions of 61,122,244, and 288µg. The gyroscopes have full-scale ranges of ±250, 500, 1000, and 2000 dps and resolutions of 0.008,0.015,0.030, and 0.061 dps. Magnetometers have a full-scale range of ±4900µT and a resolution of 0.075µT.

### 5.2. Spirit1

Spirit1 is a low-data-rate and low-power sub-1GHz transceiver. The SPIRIT1 is a highly energy-efficient RF transceiver designed for wireless applications in the sub-1 GHz frequency range. It operates in ISM and SRD bands at the frequencies of 169 MHz, 315 MHz, 433 MHz, 868 MHz, and 915 MHz. It can also be configured to operate at other frequencies in the 300–348 MHz, 387–470 MHz, and 779–956 MHz bands. The air data rate of the SPIRIT1 is adjustable and can range from 1 kbps to 500 kbps. Table 3 presents the power consumption in each mode, the latency, and the description for each mode. Our spirit1 operates at 868 MHz with a transmission power of 0 dBm.

### 5.3. Received Signal Strength Indicator (RSSI)

**Figure 4:**
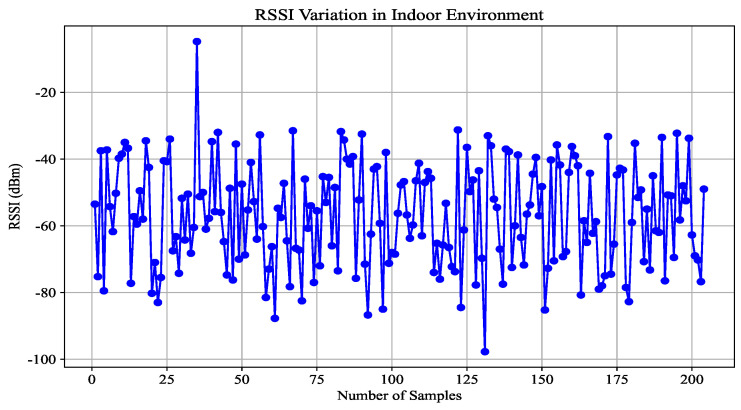
RSSI variation in an indoor environment.

The Received Signal Strength Indicator (RSSI) is a metric in wireless communications that measures the strength of a radio signal. In indoor positioning, we can measure the intensity of the RSSI signal to estimate the distance between a mobile device and a fixed infrastructure such as Wi-Fi or Bluetooth. Although cost-effective and accurate for large-scale applications, RSSI can be unstable due to interference and environmental factors, such as multipath fading, affecting reliability even in controlled indoor settings [29]. Figure 4 presents a variation of the RSSI in an indoor environment versus distances. Equations (7) and (8) present the path loss model of RSSI and the extracted distance.

(7) $$\mathrm{RSSI}\left(d\right)=\mathrm{RSSI}\left(d_{0}\right)-10n\log_{10}\left(\frac{d}{d_{0}}\right)+X_{\sigma }$$

(8) $$d_{\mathrm{RSSI}}=10^{\frac{\left(RSSI_{\mathrm{ref}}-RSSI\right)}{10\eta }}$$

where *d* represents the distance between the transmitter and the receiver device, while $d_{0}$ is the reference distance. The term RSSI$\left(d_{0}\right)$ denotes the Received Signal Strength Indicator (RSSI) value measured by the device at the reference distance $d_{0}$. Additionally, $X_{\sigma }$ refers to the Gaussian-distributed noise with a mean of zero and a variance of $\sigma^{2}$. The path loss exponent is denoted by η, which characterizes how signal strength diminishes with environmental factors. Among the various techniques used for indoor positioning, Received Signal Strength Indication (RSSI) is one of the most widely used methods due to its low cost. However, distance estimation using the RSSI path loss model remains a challenging task, especially in complex indoor environments. An accurate determination of critical parameters, including the RSSI distribution and the path loss exponent η, is vital in improving positioning accuracy. This is particularly important in non-line-of-sight (NLOS) conditions, where environmental factors significantly affect the characteristics of the wireless communication channel. Overcoming these challenges can lead to significant improvements in the accuracy of indoor positioning systems [6]. Figure 4 shows an example of RSSI values obtained from more than 200 repeated measurements for a fixed distance between two sensor nodes, wherein we can observe the variation in the measurement, which causes the problem of measurement accuracy and significantly affects the precision of the predicted distance.

### 5.4. Environment Setup

**Figure 5:**
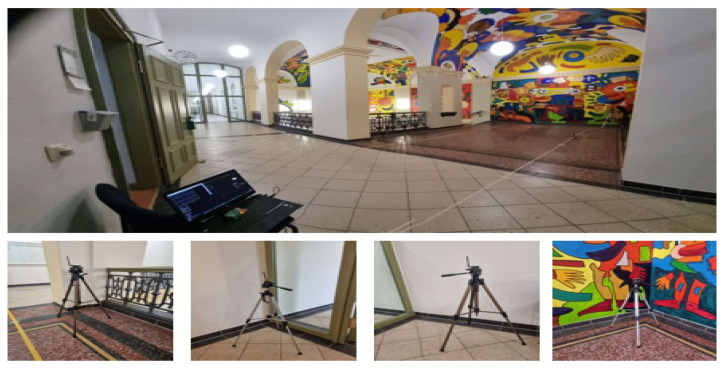
Real measurement environment.

The database used to train our AI models was obtained from various measurements taken in a real environment that included obstacles with variable temperature and humidity, which affected the path loss coefficient η. In this section, we describe the measurement environment used. The experiment was conducted on a single floor of a building with approximate dimensions of 9.5 m by 12.5 m. Figure 5 illustrates the real measurement environment. For the subsequent tests, four different anchors were used to collect the data measurements for different positions and through the mobility of the target. These anchors were strategically placed in a corridor at a height of 1.5 m above the floor, forming a two-dimensional (x, y) plane, as detailed in Table 4. Additional environmental parameters are provided in Table 5.

### 5.5. Data Acquisition

The dataset comprises ID, RSSI, x, and y of each anchor and measurements from the ICM-20948 sensor 9-axis and spirit1. The data offer comprehensive information about each point, along with spatial orientation data derived from the 9-axis measurements and signal strength details obtained from the RSSI measurements. We used the data conversion formulas shown in Equation (9) to convert the raw sensor outputs into meaningful measurements. We divided the output by its sensitivity scale factor to obtain the converted data [30]:

(9) Converteddata=output/sensitivityscalefactor

### 5.6. Data Preprocessing

Data preprocessing is a crucial phase in preparing datasets for machine learning and deep learning models, ensuring that the data are clean, consistent, and ready for analysis. This phase includes several essential techniques to improve data quality and model performance. Data cleaning is fundamental and includes the manipulation of missing values through imputation or removal, and the elimination of redundant or irrelevant features. Missing values are handled by first identifying them and then choosing between imputation methods such as mean or median, or dropping rows or columns with large amounts of missing data. Redundant features, which are either highly correlated with others or do not provide any additional value, should be removed to enhance the efficiency of the models.

Scaling and normalization are critical preprocessing steps to ensure consistency across datasets, especially for models that are sensitive to the scale of input features. Feature engineering, which includes techniques such as coding categorical variables and generating interaction terms, can further improve model performance. Standardization involves calculating the mean (μ) and standard deviation (σ) for each characteristic or column in the dataset. The data are then centered around 0 by subtracting the mean (μ) from each value, and scaled by dividing each centered value by the standard deviation (σ). This process results in a dataset where each feature has a mean of 0 and a standard deviation of 1. Mathematically, the formula for standardizing a variable *x* is given by (10):

(10) $$z=\frac{x-\mu }{\sigma }$$

where *x* is the original value, μ is the mean of the feature, σ is the standard deviation of the feature, and *z* is the standardized value. This process ensures that each feature contributes equally to the analysis or modeling process [31].

### 5.7. Cross-Validation with Grid Search

**Figure 6:**
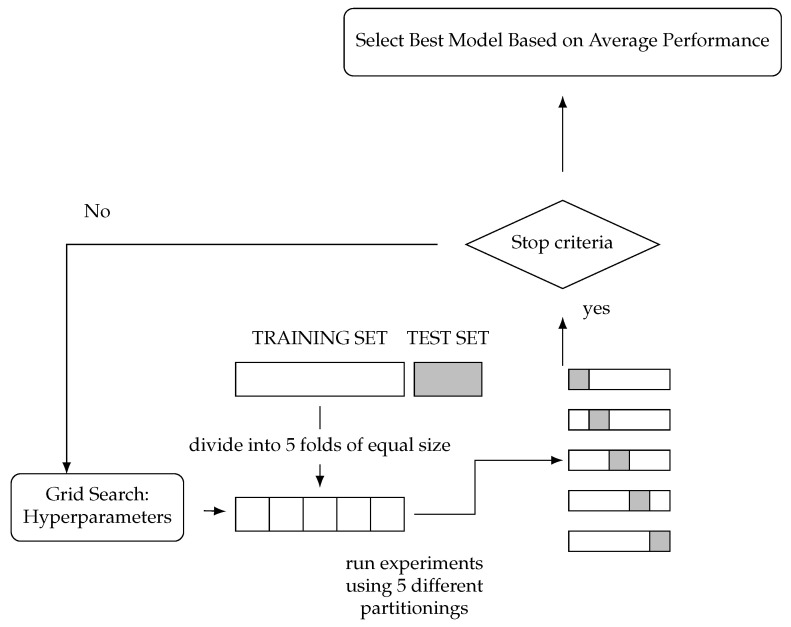
K-fold cross-validation process with grid search.

The accuracy value can be optimized by using grid search cross-validation. Grid search is the process of selecting a combination of models and hyperparameters by testing the combinations one by one and validating each combination. The goal of grid search is to determine the combination that produces the best model performance that can be selected to be used as a model for prediction [32]. Grid search is usually combined with k-fold cross-validation, which provides an evaluation index for the classification model. The k-fold cross-validation process is detailed in Figure 6.

## 6. Comparative Analysis Strategy of AI Models

### 6.1. First Scenario

Our first process involved data acquisition, where various sensors, including IMU sensors such as the ICM-20948, were used to collect 9-axis measurements and RSSI data, collecting crucial information on orientation, acceleration, and magnetic fields, among others.

**Figure 7:**
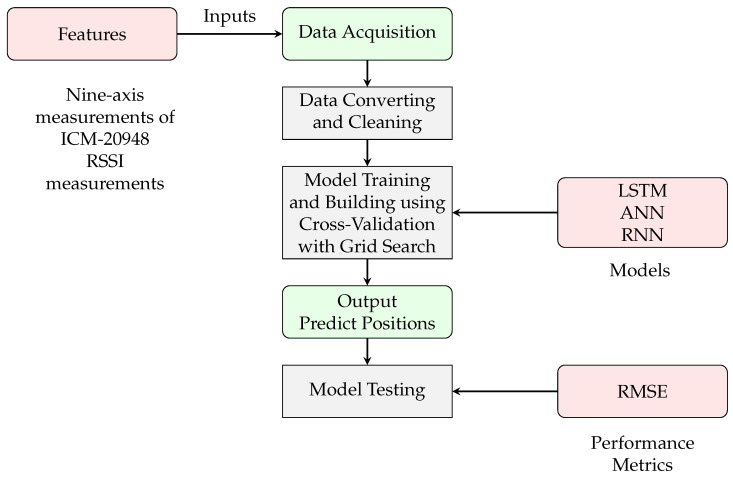
Flowchart of the proposed algorithm using the first scenario.

The acquired data were then split into test data, which were used to evaluate the model’s performance. These split data were then used for model testing, with a portion used to train the models and the remainder reserved for testing. Typically, a 70-20-10 split is used, where 70 percent of the data are used for training, 20 percent are used for testing, and 10 percent are used for validation. However, before diving into model training, data cleansing and conversion with ICM-20948 calibration was essential. This involved sorting through the data and ensuring that they were formatted in a way that the models could understand. Data conversion formulas were used to translate raw sensor outputs into meaningful measurements. Through a combination of cross-validation and grid search, we optimized the models using a variety of deep learning algorithms to find the best combination with the least error. All the parameters used for the ANN, RNN, and LSTM are presented in Table 6 and Table 7, respectively. The flowchart of the algorithm is presented in Figure 7.

Hyperparameters play a crucial role in determining the performance of Recurrent Neural Networks (RNNs), Long Short-Term Memory (LSTM) networks, and Artificial Neural Networks (ANNs). The hyperparameters for three different neural network models are detailed, providing insight into their configurations and training setups. Table 6 shows the hyperparameters for the Artificial Neural Network (ANN) and the Recurrent Neural Network (RNN) models. They both use the Rectified Linear Unit (ReLU) as their activation function, chosen for its efficiency in deep network training. The ADAM optimizer, known for its adaptive learning rate capabilities, is used with the Mean Squared Error (MSE) as the loss function due to its effectiveness in regression tasks. The network architecture includes a 13-neuron input layer and a variable number of neurons in the hidden layers—specifically, 8, 16, and 32 neurons—in different configurations of one, two, or three hidden layers. The output layer has two neurons. Training is performed with a batch size of 32 over 50 epochs.

Table 7 details the hyperparameters for the Long Short-Term Memory (LSTM) model. Unlike the ANN and RNN, the LSTM model integrates LSTM units, with options for 50 or 100 units and 16 or 32 dense units within a single LSTM layer. The activation function and optimizer remained as ReLU and ADAM, respectively, and MSE was used as the loss function. The LSTM model also had an output layer with two neurons, a batch size of 32, and training over 50 epochs. Also, a timestep of 1 was specified, which reflected the length of the input sequences processed during training.

### 6.2. Second Scenario

**Figure 8:**
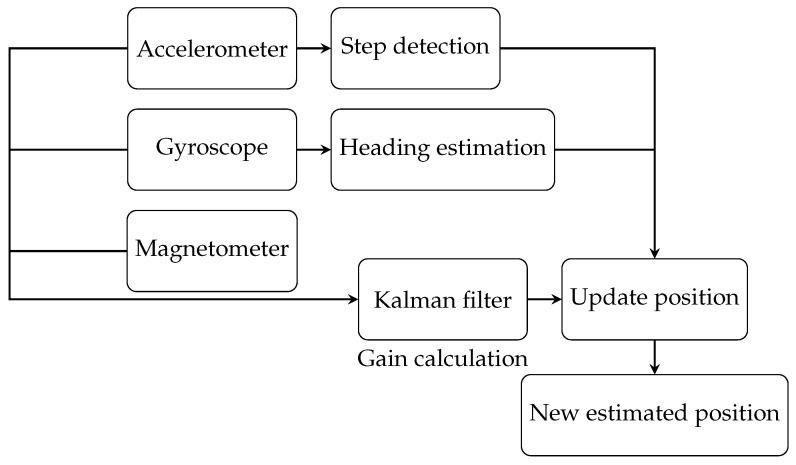
Flowchart of the proposed algorithm for the second scenario.

In the second scenario, we used a Kalman filter with heading estimation to determine the position of the target. The position estimation process is shown in Figure 8. This estimation is based solely on processed 9-axis measurements from the ICM-20948 sensor, which integrates data from the accelerometer, magnetometer, and gyroscope. The Kalman filter gain was calculated using these 9-axis measurements. Since our localization is in 2D, only the x- and y-axis values were used.

**Figure 9:**
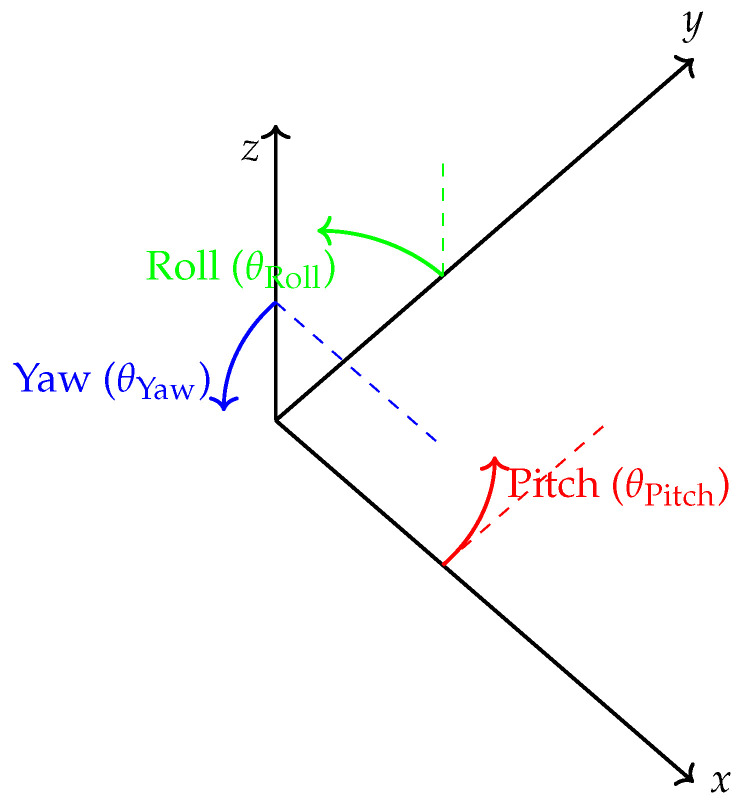
Euler angles.

For step detection, we used pitch and roll angles derived from Euler angles to estimate step length. Our target is stationary. Euler angles are defined in Figure 9. In this context, pitch refers to rotation around the x-axis, roll refers to rotation around the y-axis and yaw refers to rotation around the z-axis. To calculate the pitch and roll angles, we used Equations (11) and (12) [33]:

(11) $$\mathrm{pitch}=\arctan\left(\frac{-a_{x}}{\sqrt{a_{y}^{2}+a_{z}^{2}}}\right)$$

(12) $$\mathrm{Roll}=\arctan2(a_{y},a_{x})$$

Pitch and roll angles calculated from the accelerometer data remained stable over time because they were based on measurements of the Earth’s gravity rather than integration. In addition, low-cost accelerometers generally have better characteristics such as lower noise levels and greater stability than low-cost gyroscopes. Using the pitch and roll, we calculated the step length along the x-axis and y-axis. Equations (13) and (14) show the step length along to x-axis and y-axis:

(13) step_length_x=cos(pitch)

(14) step_length_y=sin(roll)

Another critical calculation of position estimation is heading. Heading estimation refers to the process of determining the direction of movement of the target. Accurate heading information is essential for assessing and predicting the target path. For heading calculation, we used the gyroscope measurements. The heading is presented in Equation (15):

(15) $$\mathrm{Heading}=arctan(g_{x}/g_{y})$$

After the heading calculation, we used Equations (16) and (17) to calculate the position of $x_{target}$ and $y_{target}$:

(16) $$X_{t}=X_{t-1}+SL\;\cdot \;\cos\left(\mathrm{Heading}\right)$$

(17) $$Y_{t}=Y_{t-1}+SL\;\cdot \;\sin\left(\mathrm{Heading}\right)$$

where SL are step_length_x and step_length_y. $X_{t-1}$ and $Y_{t-1}$ are the updated positions using the Kalman filter. The initial position is defined as input.

### 6.3. Third Scenario

**Figure 10:**
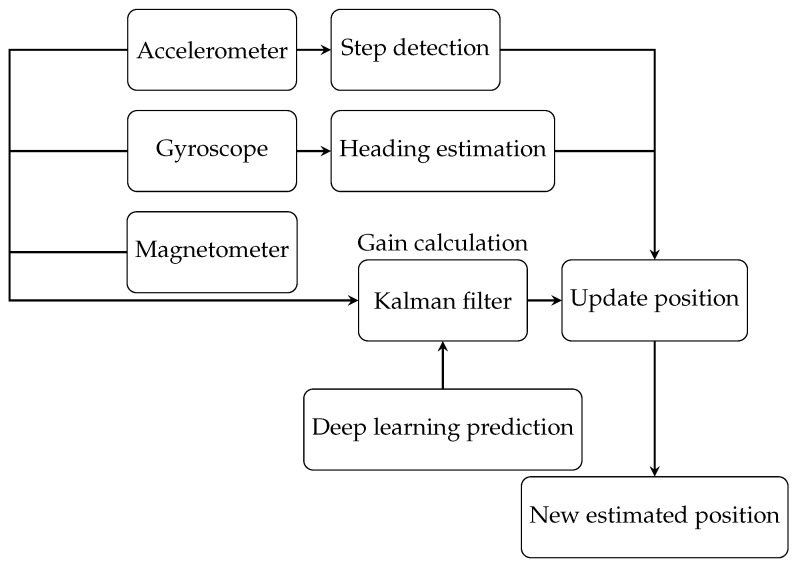
Flowchart of the proposed algorithm with deep learning integration.

In the third scenario, the proposed approach integrated a neural network model with a Kalman filter to improve system performance. Figure 10 shows the flowchart of the proposed scenario. The Kalman filter played a dual role in predicting the system state and refining it through an update step. In this hybrid approach, deep learning was used to estimate the position and the Kalman filter was used to correct it.

This combination allowed the neural network to use its predictive capabilities to provide an initial estimate for each position, while the Kalman filter incorporated sensor data from accelerometers, gyroscopes, and magnetometers for refinement. Importantly, the Kalman filter continued to independently compute pitch, roll, and heading angles using conventional methods without direct involvement of the neural network. The neural network models used in this analysis included a Recurrent Neural Network (RNN), Artificial Neural Network (ANN), and Long Short-Term Memory (LSTM) network.

## 7. Results and Discussion

This research provides an in-depth comparison of different models based on error metrics and processing time, with a special focus on mobility scenarios. In order to reduce localization delays, we conducted two different studies: one using grid search and cross-validation to identify the optimal models for LSTM, RNN, and ANN, and another aimed at selecting the best features to minimize the error in the output coordinates. We present the results of three scenarios. In the first scenario, cross-validation combined with grid search is used to identify the optimal hyperparameter tuning. Then, feature selection is applied to evaluate both accuracy and processing time. The selected results from this scenario are then used as input to the Kalman filter in the third scenario. We used several performance measures, including Root Mean Square Error (RMSE), Mean Absolute Error (MAE), and R-squared, to evaluate and characterize the effectiveness of our models. The RMSE is defined by Equation (18):

(18) $$RMSE=\frac{1}{n}\sum_{i=1}^{n}{\left(y_{i}-{\hat{y}}_{i}\right)}^{2}$$

where *n* is the total number of data points, $y_{i}$ is the observed (actual) value for data point *i*, and ${\hat{y}}_{i}$ is the predicted value for the data point *i*. The MAE is defined in Equation (19):

(19) $$\mathrm{MAE}=\frac{1}{n}\sum_{i=1}^{n}\left|y_{i}-{\hat{y}}_{i}\right|$$

where $y_{i}$ are the actual values, ${\hat{y}}_{i}$ are the predicted values, and *n* is the number of samples.

The R-squared ($R^{2}$) is presented in Equation (20):

(20) $$R^{2}=1-\frac{SS_{\mathrm{res}}}{SS_{\mathrm{tot}}}$$

where $SS_{\mathrm{res}}$ is the sum of squares of residuals and $SS_{\mathrm{tot}}$ is the total sum of squares.

In deep learning, effective feature selection is critical for enhancing model performance. Recursive Feature Elimination (RFE) is a useful technique for this, especially for complex models like Recurrent Neural Networks (RNNs) and Long Short-Term Memory (LSTM) Networks. RFE works by recursively removing the least important features and retraining the model to identify the most significant features [34]. Feature selection is crucial for improving short-term load forecasting. Although increasing the number of features can enhance prediction performance, using too many features may introduce irrelevant or non-informative features, potentially degrading predictive accuracy. Irrelevant features do not provide useful information and can confuse the learning algorithm, highlighting the need for effective feature selection.

Another method, exhaustive feature selection, evaluates all possible feature combinations to find the optimal subset. While thorough, this approach is computationally expensive and less practical for a large number of features. Combining RFE with other methods, such as model-based feature importance from Random Forests, can refine the selection process when many features have similar importance. It is also essential to monitor for overfitting, which can occur if too many features are selected, potentially degrading performance on unseen data [35].

In our analysis, we used a Random Forest model combined with Recursive Feature Elimination (RFE) to identify the most important features for predicting the x and y coordinates. We use Random Forest to select the best 7 features with respect to the outputs that are $x_{target}$ and $y_{target}$. Our Random Forest model uses 100 trees and 7 as the maximum depth. The algorithm uses for each tree a subset of features and calculates its average score over all trees using the selected feature. The results are presented in Table 8, highlighting the top seven features selected from RSSI, 9-axis measurements, ID, and the x and y anchor coordinates. The selected features are mx, gz, my, ax, RSSI, mz, and gx. Table 8 presents a ranking of features based on their importance values. The highest-ranked feature is mx with an importance score of 0.4889, followed by gz and my with scores of 0.2418 and 0.2271, respectively. The remaining features have significantly lower importance values, with ax ranking fourth with 0.0333, and RSSI and mz trailing with much smaller values. The feature gx has the lowest rank with an importance of 0.0008.

**Figure 11:**
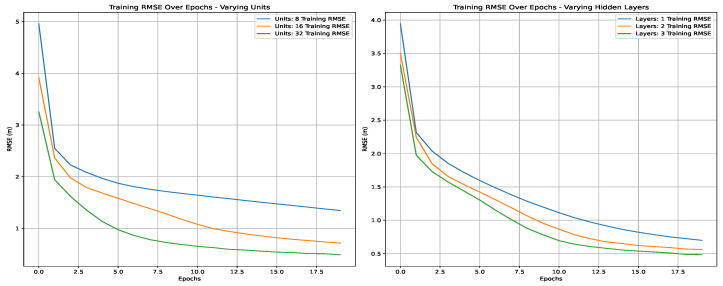
Training RMSE over epochs using an ANN.

**Figure 12:**
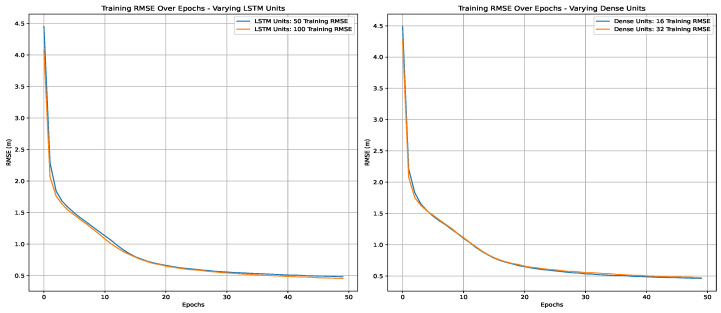
Training RMSE over epochs using LSTM.

**Figure 13:**
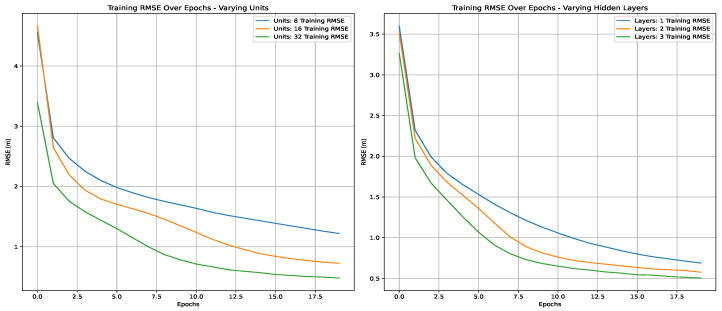
Training RMSE over epochs using an RNN.

Using the cross-validated grid search in the first scenario, we tried to find the best hyperparameters for the studied three deep learning techniques. Figure 11, Figure 12 and Figure 13 present the variations in RMSE through the epochs for different hyperparameters. The best parameters are summarized in Table 9. The LSTM model has one layer with 100 units and 32 dense units. Both the RNN and ANN models have three layers with 32 units.

The RMSE was reduced by 0.01 m for the ANN, 0.02 m for the RNN, and 0.03 m for the LSTM by applying the feature selection results to each model. Table 10, Table 11 and Table 12 show the performance metrics of the optimized models. Performance metrics of the optimized models where the hyperparameters were fine-tuned using grid search and cross-validation techniques. The RMSE, $R^{2}$, MAE, total time, and time per observation are reported for each model. The results are given for both the validation and test datasets.

Feature selection had varying effects on the accuracy and computational efficiency of each model when comparing the performance of the ANN, RNN, and LSTM models. A key metric, Root Mean Square Error (RMSE), improved across all models after feature selection was applied.

For the ANN, the RMSE decreased from 0.47 m to 0.46 m on the validation dataset and from 0.44 m to 0.40 m on the test dataset. The RNN showed the most significant improvement, with the validation RMSE dropping from 0.43 m to 0.41 m and the test RMSE dropping from 0.44 m to 0.38 m, which is the largest reduction among the three models. The LSTM model showed an improvement from 0.54 m to 0.51 m and a reduction in the test RMSE from 0.51 m to 0.49 m. These results indicate that the RNN model is the most efficient for feature selection in terms of error reduction, with ANN tracking and LSTM showing the highest error among the three models.

For the $R^{2}$ resolution, both the RNN and the LSTM models show an improvement in the feature selection, while the ANN remains relatively robust. $R^{2}$ for the RNN improved from 0.97 to 0.98 on the validation dataset and stabilized at 0.97 on the test dataset. Similar improvements were observed for the LSTM, where $R^{2}$ increased from 0.96 to 0.97 on both datasets, indicating better model performance after feature selection. However, the ANN had a slight decrease in the test dataset, where $R^{2}$ decreased from 0.98 to 0.97, although it remained constant at 0.98 on the validation dataset. This indicates that both RNN and LSTM benefit from feature selection throughout model fitting, while ANN can show small differences.

The prediction time per sample shows a slight increase for RNN, with an additional 0.01 s. These changes increase the similarity in time prediction among the models. Overall, RNN proves to be the best model, providing the fastest execution time and significant error reduction.

To analyze the performance of the different models, we introduced errors into the validation and test datasets, focusing on the 70% and 90% error averages. For the 70% mean error, the Recurrent Neural Network (RNN) outperforms the other models, with an error reduction of 0.04 m compared to the Artificial Neural Network (ANN) and 0.14 m compared to the Long Short-Term Memory (LSTM) in models using feature selection.

For the 90% mean error, the RNN continues to show superior performance with a mean error of 0.43 m compared to 0.48 m for ANN and 0.67 m for LSTM. These results highlight the robustness of the RNN in dealing with higher levels of error compared to the ANN and LSTM models. The analyses using the different models are presented in Table 13.

**Figure 14:**
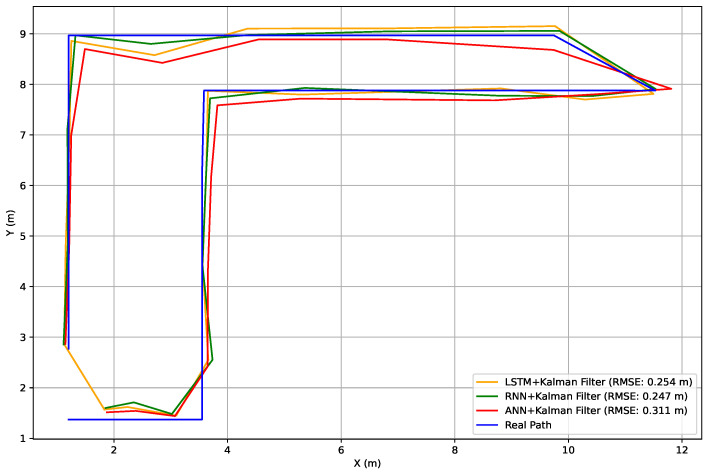
Comparison of actual and predicted path using different models.

For the second scenario, we used the Kalman filter for the 9-axis of the ICM-20948. The proposed algorithm achieves a Root Mean Square Error (RMSE) of 3.34 m. In the third scenario, by integrating the Kalman filter with deep learning models, we observe improved performance compared to the first and second scenarios. Prior to this combination, the RMSE values for the ANN, RNN, and LSTM were 0.46 m, 0.41 m, and 0.51 m, respectively. After applying the hybrid solution in the third scenario, the RMSE using the predicted positions from the ANN decreases to 0.311 m, while the RMSE using predictions from the RNN and LSTM reduces to 0.247 m and 0.254 m, respectively. Figure 14 presents a comparison between the real path and the hybrid models.

**Figure 15:**
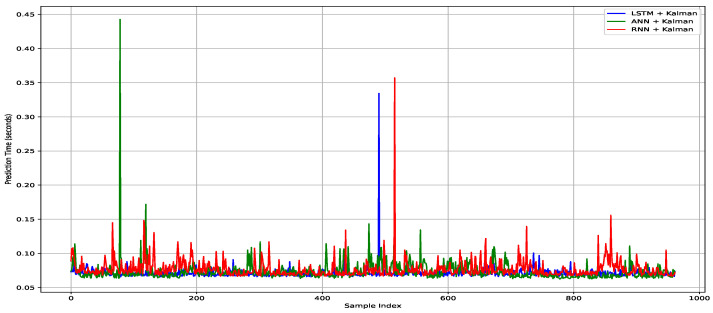
Prediction time combining Kalman filter with models.

**Figure 16:**
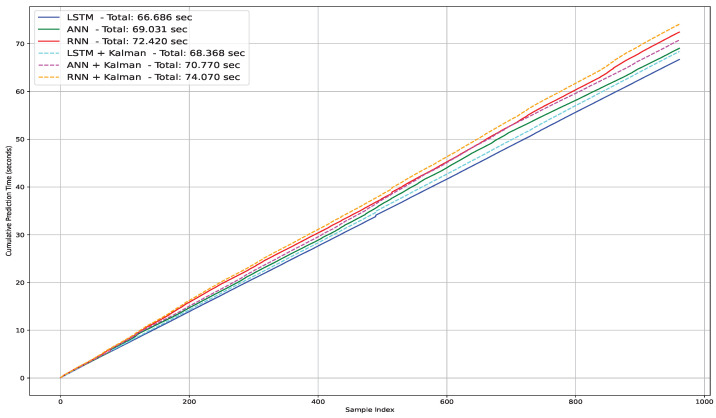
Comparison of prediction time.

Using the hybrid models, the prediction per sample was still relatively the same. Figure 15 presents the prediction time for hybrid models. By combining the Kalman filter with deep learning models, the total prediction time increased from 66.686 s to 68.368 s when using the LSTM with the Kalman filter. For the RNN and ANN combined with the Kalman filter, the prediction times increased to 74.070 s and 70.770 s, respectively. The predictions for each sample remained stable at 0.077 s, 0.074 s, and 0.071 s for the RNN, ANN, and LSTM models combined with the Kalman filter, respectively. Figure 16 presents a comparison between the hybrid models and the classic models.

The total time is calculated for 962 samples. Increasing the number of predicted positions highlights the difference between the first and third scenarios, where the RMSE decreases by an average of 0.2 m. This change may indicate a delay in mobility tasks. After analyzing the results, the combination of RNN with the Kalman filter demonstrates the best performance. RNN combined with Kalman filter presents an RMSE of 0.247 m.

Compared to other research results, our model focuses on errors in both localization and time processing. Table 14 presents a comparison between our results and those recently published. By integrating the Kalman filter with the RNN algorithm, we achieved a localization error of 0.247 m, significantly lower than the approximately 1 m error commonly reported in the literature. Despite the measurement uncertainties inherent to the RSSI technique, our AI model demonstrated the ability to predict distances with greatly reduced uncertainty. Additionally, unlike other studies, we prioritized minimizing processing time, achieving a detection time of no more than 77 ms for the position of our mobile target. This achievement makes real-time localization of mobile targets possible.

## 8. Conclusions

This research successfully conducted a comparative analysis of several AI models, including Artificial Neural Networks (ANNs), Recurrent Neural Networks (RNNs), Long Short-Term Memory (LSTM), and Kalman filters, aimed at enhancing the accuracy of indoor positioning systems. By integrating data from a 9-axis ICM-20948 sensor with RSSI measurements, the study provided valuable insights into model performance for real-time localization. Among the models, the RNN consistently demonstrated superior performance, achieving a localization error as low as 0.36 m with minimal processing delay. Additionally, combining deep learning models with Kalman filtering significantly improved performance, reducing the Root Mean Square Error (RMSE) to 0.247 m and enhancing tracking accuracy, particularly in dynamic and noisy indoor environments. In our case study, the mobile node sends the three detected positions of the three closest nodes, and the localization of the mobile target is calculated in the cloud. To further accelerate the computation time, we plan to use edge computing and integrate the calculations into suitable hardware [36]. Processing time plays a critical role in extending the lifetime of a network, especially in resource-constrained environments such as wireless sensor networks and IoT systems. Efficient processing reduces the workload on individual nodes, minimizing their energy consumption and delaying battery depletion. However, adopting advanced techniques such as edge computing, while significantly improving processing efficiency and latency, can pose challenges due to its high energy consumption. To address this, future work will focus on integrating energy-harvesting solutions that enable nodes and edge devices to generate power from renewable sources such as RF energy harvesting. This approach ensures sustainable energy availability, balancing the benefits of edge computing with the need for extended network life and continuous operation.

## Figures and Tables

**Table 1:** Performance overview of machine learning algorithms.

Reference	ML Algorithms	Technologies	Techniques	Tested Area	Error
**[17]**	FNN	BLE	RSSI	$30\;m^{2}$	1.0m
**[18]**	KNN	WiFi	RSSI	5m×6m	1.07m
**[19]**	RF	UWB	RSSI	9.6m×7.9m	1.0m
**[20]**	MLP	ZigBee	RSSI	5.7m×8.2m	0.95m

**Table 2:** Position of anchors in a real environment.

Characteristic	Accelerometers	Gyroscopes	Magnetometers
Full-Scale Range	±2, 4, 8, 16 g	±250, 500, 1000, 2000 dps	±4900µT
Resolution	61,122,244,288µg	0.008,0.015,0.030,0.061 dps	0.075µT

**Table 3:** SPIRIT1 transceiver states and power consumption.

State	Power Consumption (mA)	Latency (ms)	Description
Standby	1.3	0.5	Low power mode with all circuits off
Sleep	1	5	Lowest power mode
Ready	7	-	Crystal oscillator on, fast switching between states
RX Mode	11.5	-	Active reception of data, varies with data rate
TX Mode (0 dBm)	21	-	Active transmission at 0 dBm output power
TX Mode (+11 dBm)	38	-	Active transmission at +11 dBm output power
Shutdown	0.1	7	All circuits off, memory retained

**Table 4:** Position of anchors in a real environment.

Anchor Number	Positions (x, y)
2	(0.606 m, 9.154 m)
3	(0.606 m, 0.588 m)
4	(4.062 m, 5.711 m)
5	(11.489 m, 8.967 m)

**Table 5:** Environment setup.

Parameters	Values
Areas	12.5 m × 9.5 m
Anchor number	4

**Table 6:** Hyperparameters used in ANN and RNN models.

Parameters	Values
Activation Function	ReLu
Optimizer	ADAM
Loss	MSE
Number of neurons in input layer	13
Number of neurons in hidden layers	8, 16, 32
Number of hidden layers	1, 2, 3
Number of neurons in output layer	2
Batch size	32
Number of epochs	50

**Table 7:** Hyperparameters and settings used in the LSTM model.

Parameters	Values
Activation Function	ReLU
Optimizer	ADAM
Loss Function	MSE
Number of LSTM Units	50, 100
Number of Dense Units	16, 32
Number of Hidden Layers	1 (LSTM layer)
Number of Neurons in Output Layer	2
Batch Size	32
Number of Epochs	50
Timesteps (for LSTM)	1

**Table 8:** Feature importances ranked by their importance values.

Feature	Rank	Importance
mx	1	0.4889
gz	2	0.2418
my	3	0.2271
ax	4	0.0333
RSSI	5	0.0031
mz	6	0.0026
gx	7	0.0008

**Table 9:** Grid search with cross-validation results.

Model	Layers	Units	Dense Units
LSTM	1	100	32
RNN	3	32	-
ANN	3	32	-

**Table 10:** Performance metrics for different datasets using an ANN.

Dataset	Metric	Values Without Feature Selection	Values with Feature Selection
Validation Dataset	RMSE	0.47 m	0.46 m
	MAE	0.24 m	0.24 m
	$R^{2}$	0.98	0.98
	Prediction Time	61.55 s	69.03 s
	Time per Observation	0.06 s	0.07 s
Test Dataset	RMSE	0.44 m	0.40 m
	MAE	0.22 m	0.23 m
	$R^{2}$	0.98	0.97
	Prediction Time	122.92 s	131.43 s
	Time per Observation	0.06 s	0.07 s

**Table 11:** Performance metrics for different datasets using RNN.

Dataset	Metric	Values Without Feature Selection	Values with Feature Selection
Validation Dataset	RMSE	0.43 m	0.41 m
	MAE	0.21 m	0.21 m
	$R^{2}$	0.97	0.98
	Prediction Time	62.97	72.42 s
	Time per Observation	0.07 s	0.08 s
Test Dataset	RMSE	0.44 m	0.38 m
	MAE	0.22 m	0.20 m
	$R^{2}$	0.97	0.97
	Prediction Time	127.69 s	146.44 s
	Time per Observation	0.07 s	0.08 s

**Table 12:** Performance metrics for different datasets using LSTM.

Dataset	Metric	Values Without Feature Selection	Values with Feature Selection
Validation Dataset	RMSE	0.54 m	0.51 m
	MAE	0.32 m	0.30 m
	$R^{2}$	0.96	0.97
	Prediction Time	63.08 s	66.69 s
	Time per Observation	0.07 s	0.07 s
Test Dataset	RMSE	0.51 m	0.49 m
	MAE	0.30 m	0.30 m
	$R^{2}$	0.96	0.97
	Prediction Time	128.10 s	142.58 s
	Time per Observation	0.07 s	0.07 s

**Table 13:** Performance metrics for different datasets using feature selection.

Models	Dataset	Average of 70% Errors (m)	Average of 90% of Errors (m)
ANN	Test	0.31	0.48
	Validation	0.33	0.51
RNN	Test	0.27	0.43
	Validation	0.29	0.47
LSTM	Test	0.41	0.67
	Validation	0.43	0.68

**Table 14:** Comparison of our work results with the state of the art in different scale areas overview of machine learning algorithms.

Reference	ML Algorithms	Technologies	Techniques	Tested Area	Error
**[17]**	FNN	BLE	RSSI	$30\;m^{2}$	1.0m
**[18]**	KNN	WiFi	RSSI	5m×6m	1.07m
**[19]**	RF	UWB	RSSI	9.6m×7.9m	1.0m
**[20]**	MLP	ZigBee	RSSI	5.7m×8.2m	0.95m
This work	RNN + Kalman filter	-	RSSI + 9-axis	12.5m×9.5m	0.247 m

## Data Availability

Data are contained within the article.
